# Immunoproteomics and Surfaceomics of the Adult Tapeworm *Hymenolepis diminuta*

**DOI:** 10.3389/fimmu.2018.02487

**Published:** 2018-11-12

**Authors:** Daniel Młocicki, Anna Sulima, Justyna Bień, Anu Näreaho, Anna Zawistowska-Deniziak, Katarzyna Basałaj, Rusłan Sałamatin, David Bruce Conn, Kirsi Savijoki

**Affiliations:** ^1^Department of General Biology and Parasitology Medical University of Warsaw, Warsaw, Poland; ^2^Witold Stefański Institute of Parasitology Polish Academy of Sciences, Warsaw, Poland; ^3^Department of Veterinary Biosciences University of Helsinki, Helsinki, Finland; ^4^Department of Parasitology and Vector-Borne Diseases National Institute of Public Health–National Institute of Hygiene, Warsaw, Poland; ^5^Department of Invertebrate Zoology, Museum of Comparative Zoology, Harvard University Cambridge, MA, United States; ^6^One Health Center, Berry College Mount Berry, GA, United States; ^7^Division of Pharmaceutical Biosciences University of Helsinki, Helsinki, Finland

**Keywords:** *Hymenolepis diminuta*, tapeworm, cestoda, host–parasite interactions, proteomics, mass spectrometry, immunoblotting, surface proteins

## Abstract

In cestodiasis, mechanical and molecular contact between the parasite and the host activates the immune response of the host and may result in inflammatory processes, leading to ulceration and intestinal dysfunctions. The aim of the present study was to identify antigenic proteins of the adult cestode *Hymenolepis diminuta* by subjecting the total protein extracts from adult tapeworms to 2DE immunoblotting (two-dimensional electrophoresis combined with immunoblotting) using sera collected from experimentally infected rats. A total of 36 protein spots cross-reacting with the rat sera were identified using LC-MS/MS. As a result, 68 proteins, including certain structural muscle proteins (actin, myosin, and paramyosin) and moonlighters (heat shock proteins, kinases, phosphatases, and glycolytic enzymes) were identified; most of these were predicted to possess binding and/or catalytic activity required in various metabolic and cellular processes, and reported here as potential antigens of the adult cestode for the first time. As several of these antigens can also be found at the cell surface, the surface-associated proteins were extracted and subjected to in-solution digestion for LC-MS/MS identification (surfaceomics). As a result, a total of 76 proteins were identified, from which 31 proteins, based on 2DE immunoblotting, were predicted to be immunogenic. These included structural proteins actin, myosin and tubulin as well as certain moonlighting proteins (heat-shock chaperones) while enzymes with diverse catalytic activities were found as the most dominating group of proteins. In conclusion, the present study shed new light into the complexity of the enteric cestodiasis by showing that the *H. diminuta* somatic proteins exposed to the host possess immunomodulatory functions, and that the immune response of the host could be stimulated by diverse mechanisms, involving also those triggering protein export via yet unknown pathways.

## Introduction

Cestodes have been recognized for many years as being among the most important human parasites, causing diseases that remain within the top health priorities in many parts of the world (1). Indeed, cestode diseases are explicit targets for control efforts, especially in developing countries (2, 3). Such diseases are emerging threats even in more developed countries, where hymenolepiasis remains among the cestode diseases that have the highest morbidity globally (4, 5).

Similarly to other cestode species the life-cycle of *Hymenolepis diminuta* is complex and involve three morphologically distinct developmental stages: the hexacanth larva, the metacestode juvenile, and the sexual adult stage (6). The hexacanth is enclosed within its oncospheral envelopes, forming the cestode egg (7) that undergoes progressive metamorphosis into the metacestode stage in the intermediate host's body tissues and/or cavities. In most of the cases, the metacestode needs to be ingested to reach the vertebrate definitive host's small intestine and grow into the adult parasite. In the intestinal lumen, developing immature and adult cestodes are exposed to the hostile intestinal environment, including digestive enzymes, immune responses, bacteria, and active peristaltic movements of the small intestine. Adult tapeworms utilize their scolex that is armed with adhesive structures (suckers), for anchoring themselves to the intestinal epithelium. In addition, juvenile, premature and adult cestodes use tegumental surface structures—microtiches—to mediate broad adherence to the intestinal epithelium. In a number of cases, this mechanical contact between the parasite and host intestinal tissue can irritate the intestinal mucosa, which may finally result in inflammatory processes leading to ulceration and intestinal dysfunctions (8). In this way parasite-derived molecules interact with the host immune system as antigens associated with three sources: excretory-secretory, surface, and tegumental proteins (9–11). Many of these molecules are proteins involved in the parasite's metabolism and survival strategies. In our previous study we identified numerous excretory-secretory proteins (ESPs), among which several were found as antigens with potential impact on the parasite–host interaction (10).

However, despite the current progress in understanding the parasite–host cross-talk mechanisms, the immunoparasitology and related proteomes of the adult cestodes have remained largely unknown. The only available data related to immunoproteomics of these organisms is based on the adult tapeworms *Echinococcus granulosus* infecting dogs (9). Comparing the number of reports regarding the immunoparasitology of cestode metacestode stages (predominantly hydatidosis), adult trematodes and nematodes, there is still a gap in our knowledge related to the adult cestode.

Human or other mammalian cestodiases are mostly caused by the metacestode juvenile stages of *Echinococcus* spp. (hydatid cysts) and *Taenia* spp. (cysticerci). Selected *Hymenolepis* species may infect humans in the adult stage, and *H. diminuta* has been extensively studied as it can be maintained in laboratory animal hosts (12). From these species, *H. diminuta* can establish successful invasion in both rodent and human hosts, and has become an important model for studying cestode-host interrelationships. However, from immunological perspectives, the adult stage of this organism has remained largely unexplored (13–23). The presence of adult tapeworms in the host intestine may also influence the function of this organ, thereby affecting the immunity and host condition. In support of this, Kosik-Bogacka et al. (24–31) have reported that *H. diminuta* had impact on ion transport, oxidative stress, the expression and/or activity of toll-like receptors and cyclooxygenases in rat intestines. Due to its low pathogenicity and immunomodulatory activity, *H. diminuta* is also considered a source of potential therapeutic molecules for treating autoimmune and inflammatory diseases activity (32–34).

In the present study, we applied 2DE immunoblotting (two-dimensional gel electrophoresis followed by immunoblotting) of *H. diminuta* proteins using antisera raised against this organism in rats to indicate antigenic proteins with potential role in adaptation and host–parasite interaction. In addition, the surface-associated proteins were identified to complement the 2DE results and to pinpoint the subcellular location of the identified antigens. Our study, besides uncovering plausible antigens in the adult cestodes, demonstrates that gel-based proteomic approach investigating individual proteins still offers an effective way for finding new candidates for immunodiagnostics and therapeutic strategies.

## Animals and methods

### Experimental animals

Healthy and pathogen-free male Lewis rats, aged 3 months, were used as definitive hosts for adult *H. diminuta*. They were kept in plastic cages in the laboratory animal facilities of the Institute of Parasitology, PAS. They were provided feed and water *ad libitum*.

### Ethics statement

This study was approved by the 3^rd^ Local Ethical Committee for Scientific Experiments on Animals in Warsaw, Poland (Permit number 51/2012, 30^th^ of May 2012).

### Cultivation of *H. diminuta* adult cestodes

Six-week-old *H. diminuta* cysticercoids were extracted from dissected *Tenebrio molitor* beetles under a microscope (magnification 100 ×). Three-month-old rats (10) were infected by voluntary oral uptake of six cysticercoids of *H. diminuta* per rat and the fecal sample direct smears were examined under a microscope (magnification 400 ×) after 5–6 weeks from the initial infection to verify the presence of adult parasites by finding eggs. Rats were euthanized with 100 mg/kg intraperitoneal tiopenthal anesthesia (Biochemie GmbH, Austria). The rat small intestines were removed immediately, adult parasites were isolated and washed up to five times with 100 mM PBS with antibiotics added (1% penicillin) to remove debris. Before protein extraction and proteomic analysis the parasitic material was stored at −80°C.

### Collection of serum from infected rats

Blood samples were collected 4–5 weeks after infection from rats, infected with *H. diminuta* and serum was separated. Sera before the infection at day 0 and from uninfected rats were used as negative controls (Supplementary Figure 1). Blood samples were collected to tubes by cardiac puncture at the time of euthanasia from heavily sedated animals. After collection, samples were allow to clot by leaving them undisturbed at room temperature for 20–25 min. The clot was removed by centrifugation (1,500 × *g* for 15 min, +4°C), serum samples were collected immediately and transferred into a clean polypropylene tube using a pipette. If not used immediately samples were stored at −80°C.

### Protein extraction

*Hymenolepis diminuta* adult worms in whole (size between 40 and 60 cm in length) were suspended in lysis buffer, containing 8 M Urea, 4% CHAPS and 40 mM Tris-base supplemented with protease inhibitor cocktail (Roche, Germany) and homogenized by sonication on ice until the suspension became clear. The homogenate was centrifuged at 15,000 × *g* at 4°C for 25 min to collect the supernatant containing soluble proteins, which were either used directly or stored at −80°C until use. The protein concentration was measured using a Spectrometer ND-1000 UV/Vis (NanoDrop Technologies, United States). Three biological replicates (three adult worms at the same age collected from different animals) taken from independent experiments were used in the present study.

### Two-dimensional gel electrophoresis (2DE)

The protein samples (150 ±10 μg from each replicate) were rehydrated overnight in 250 μl of the rehydration solution (ReadyPrep™ 2-D Rehydration Buffer, Bio-Rad, USA) with immobilized pH gradient (IPG) gel 7 cm strips having pH ranging from 4 to 7. Isoelectric focusing (IEF) was performed using a Protean IEF Cell (BioRad, United States) at 20°C as follows: 15 min at 250 V, then rapid ramping to 4,000 V for 2 h, and 4,000 V for 16,000 Vh (using a limit of 50 μA/strip). After IEF, the strips were first equilibrated for 25 min in equilibration buffer (ReadyPrep™ 2-D Starter Kit Equilibration Buffer I, Bio-Rad, USA), followed by a 25-min equilibration in the same buffer supplemented with 2.5% iodoacetamide (ReadyPrep™ 2-D Starter Kit Equilibration Buffer II). The second dimension, SDS-PAGE, was run on 12% polyacrylamide gel in the Midi-Protean Tera Cell (Bio-Rad, United States) with 200 V, for approximately 45 min. All the replica gels were run in the same conditions.

After 2DE, the proteomes were visualized using the Silver Staining Kit according to the manufacturer's protocol (Krzysztof Kucharczyk Techniki Elektroforetyczne, Poland), or the 2DE gels were used without staining for immunoblotting. The silver-stained gels were scanned with a GS-800 densitometer (Bio-Rad, United States) and quantitatively analyzed using Quantity One and PDQuest Analysis Software (Bio-Rad, United States). To minimize the risk of protein overstaining, the time used in the developing step was reduced to a minimum.

### 2DE-immunoblotting

Proteins from 2DE-gels were transferred by a wet transfer system (Bio-Rad, United States) to a nitrocellulose membrane (Bio-Rad, United States) that was then treated with sera collected from rats experimentally infected with *H*. *diminuta* diluted 1:500 in Protein-Free T20 (TBS) Blocking Buffer (Thermo Scientific, Rockford, United States) and then with anti-rat IgG-conjugated to horseradish peroxidase (1:8,000, Sigma Aldrich, United States). The blots were developed using the SuperSignal West Pico Chemiluminescent Substrate (ThermoFisher Scientific, United States) according to the provided instructions, and visualized using the GS-800 Densitometer (Bio-Rad, United States) and analyzed by the 1-D Analysis Software Quantity 1 (Bio-Rad, United States). The experiment was performed with three biological replicate samples.

### LC-MS/MS identification

Spots of interest were manually excised from the silver-stained gels and subjected to standard “in-gel digestion” procedure, in which they were first dehydrated with acetonitrile (ACN) and then reduced, alkylated, and digested with trypsin as previously described by Kordan et al. (35). Briefly, the gel pieces were first treated with 10 mM DTT in 100 mM NH_4_HCO_3_ for 30 min at 57°C, and then with 0.5 M iodoacetamide in 100 mM NH_4_HCO_3_ (45 min in the dark at room temperature). Proteins were digested overnight with 10 ng/μl trypsin in 25 mM NH_4_HCO_3_, at pH 8.5 (Promega, Madison, WI, United States) at 37°C. The resulting tryptic peptides were extracted in a solution containing 0.1% formic acid and 2% ACN.

The tryptic peptides were subjected to liquid chromatography and tandem mass spectrometry (LC-MS/MS) in the Laboratory of Mass Spectrometry, Institute of Biochemistry and Biophysics, Polish Academy of Sciences (Warsaw, Poland). Samples were concentrated and desalted on a RP-C18 pre-column (Waters, United States), and further peptide separation was achieved on a nano-Ultra Performance Liquid Chromatography (UPLC) RP-C18 column (Waters, BEH130 C18 column, 75 μm i.d., 250 mm long) of a nanoACQUITY UPLC system, using a 45-min linear acetonitrile gradient. The column outlet was directly coupled to the Electrospray ionization (ESI) ion source of the Orbitrap Velos type mass spectrometer (Thermo, United States), working in the regime of data dependent MS to MS/MS switch with HCD type peptide fragmentation. An electrospray voltage of 1.5 kV was used. Raw data files were pre-processed with Mascot Distiller software (version 2.5, MatrixScience).

The obtained peptide masses and fragmentation spectra were matched to the National Center Biotechnology Information (NCBI) non-redundant database NCBInr 20150115 (57,412,064 sequences; 20,591,031,683 residues), with a Cestoda filter (44,695 sequences) using the Mascot search engine (Mascot Server v. 2.4.1, MatrixScience). The following search parameters were applied: enzyme specificity was set to trypsin, peptide mass tolerance to ± 30 ppm and fragment mass tolerance to ± 0.1 Da. The protein mass was left as unrestricted, and mass values as monoisotopic with one missed cleavage being allowed. Alkylation of cysteine by carbamidomethylation as fixed and oxidation of methionine was set as a variable modification.

Multidimensional protein Identification Technology–type (MudPIT-type) and/or the highest number of peptide sequences, were selected. The expected value threshold of 0.05 was used for analysis, which means that all peptide identifications had a <1 in 20 chance of being a random match. Spectra derived from silver-stained gel pieces usually do not contain enough MS/MS fragmentations to calculate a meaningful FDR, therefore a Mascot score threshold of 30 or above (*p* < 0.05) was used.

### Extraction and identification of surface-associated proteins

Adult *H. diminuta* worms were first washed 3 times in sterile phosphate-buffered saline (PBS), followed by quick wash in sterile PBS with antibiotics (1% penicillin) to remove debris. Then they were washed again in sterile PBS without antibiotics and incubated in 3 ml of 1% Nonidet P-40 (NP-40 [Sigma Aldrich]) in 50 mM Ambic/AMBIC buffer for 30 min on a roller mixer at room temperature. After incubation samples were centrifuged at 15,000 × *g* and the supernatant was collected to a new tube. In order to perform mass spectrometry analysis Nonidet P-40 was removed using detergent Removal Spin Columns (Pierce) according to manufacturer's instructions. Columns were first equilibrated with 50 mM AMBIC without the detergent and then the sample was carefully applied on the column and incubated for 2 min in room temperature and eluted by centrifugation (1,000 × *g*, 2 min) to a new tube. The protein concentration was measured using a Spectrometer ND-1000 UV/Vis (NanoDrop Technologies, United States). Three replicates from the collected surface proteins were subjected to LC-MS/MS identification.

Proteins were identified by LC-MS/MS (Laboratory of Mass Spectrometry, Institute of Biochemistry and Biophysics, Polish Academy of Sciences (Warsaw, Poland) as described above. Protein solutions were subjected to standard procedure of trypsin digestion, during which proteins were reduced with 0.5 M (5 mM f.c.) TCEP for 1 h at 60°C, blocked with 200 mM MMTS (10 mM f.c.) for 10 min at RT and digested overnight with 10 μl of 0.1 ug/ul trypsin. The resulting peptide mixtures were applied in equal volumes of 20 μl to RP-18 pre-column (Waters, Milford, MA) using water containing 0.1% FA as a mobile phase and then transferred to a nano-HPLC RP-18 column (internal diameter 75 μM, Waters, Milford MA) using ACN gradient (0–35% ACN in 160 min) in the presence of 0.1% FA at a flow rate of 250 nl/min. The column outlet was coupled directly to the ion source of the Orbitrap Elite mass spectrometer (Thermo Electron Corp., San Jose, CA, United States) working in the regime of data-dependent MS to MS/MS switch with HCD type peptide fragmentation. A blank run ensuring absence of cross-contamination from previous samples preceded each analysis.

Raw data files were pre-processed with Mascot Distiller software (v. 2.6, MatrixScience, London, UK). The obtained peptide masses and fragmentation spectra were matched to the NCBInr database (167,148,673 sequences; 60,963,227,986 residues), with a *Cestoda* filter (49,619 sequences) using the Mascot Search Engine (MatrixScience, London, UK, Mascot Server 2.5). To reduce mass errors, the peptide and fragment mass tolerance settings were established separately for individual LC-MS/MS runs after a measured mass recalibration, resulting in values 5 ppm for parent and 0.01 Da for fragment ions. The rest of search parameters were as follows: enzyme, Trypsin; missed cleavages, 1; fixed modifications, Alkylation of cysteine by carbamidomethylation; oxidation of methionine was set as a variable modification. In each Mascot search, the score cutoff was determined automatically to obtain an FDR below 1%? The Decoy Mascot functionality was used for keeping FDR for peptide identifications below 1%.

### Proteome bioinformatics

The presence of potential N-terminal signal peptide cleavage site for the identified proteins was analyzed using the SignalP 4.1 tool (36). The identified proteins were classified according to their predicted molecular function, biological process, and cellular component using the UniProtKB database (http://www.uniprot.org/) and QuickGO (http://www.ebi.ac.uk/QuickGO/). Proteins with enzymatic properties were further classified according to Kyoto Encyclopedia of Genes and Genomes (KEGG) database (http://www.genome.jp/kegg/).

## Results

### 2DE of the *H. diminuta* adult-stage proteins

**Figure 1:**
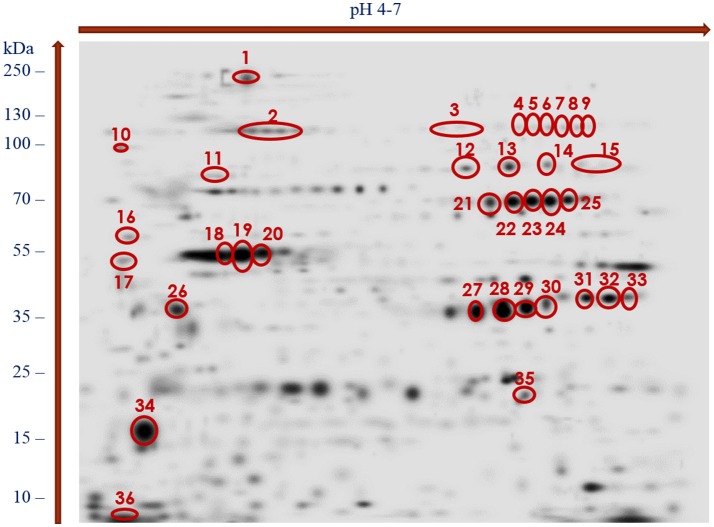
Master gel of silver-stained 2-DE protein maps of *H. diminuta* adult-stage somatic proteome showing spots recognized as immunogenic and excised from the gel for LC-MS/MS analysis (indicated by red color).

The stained 2DE-protein patterns in each three biological replica gels were comparable. As our preliminary 2DE analyses indicated that most of the adult-stage proteins migrated with pI values ranging from 4 to 7 (data not shown), the present study focused on proteins covering this proteome region. The PDQuest software analyses of the silver-stained proteomes enabled us to distinguish more than 580 adult-stage protein spots from *H*. *diminuta*. Figure 1 showing the representative silver-stained master gel indicates that majority of the protein spots migrated with molecular weights (MWs) between 15 and 130 kDa.

**Figure 2:**
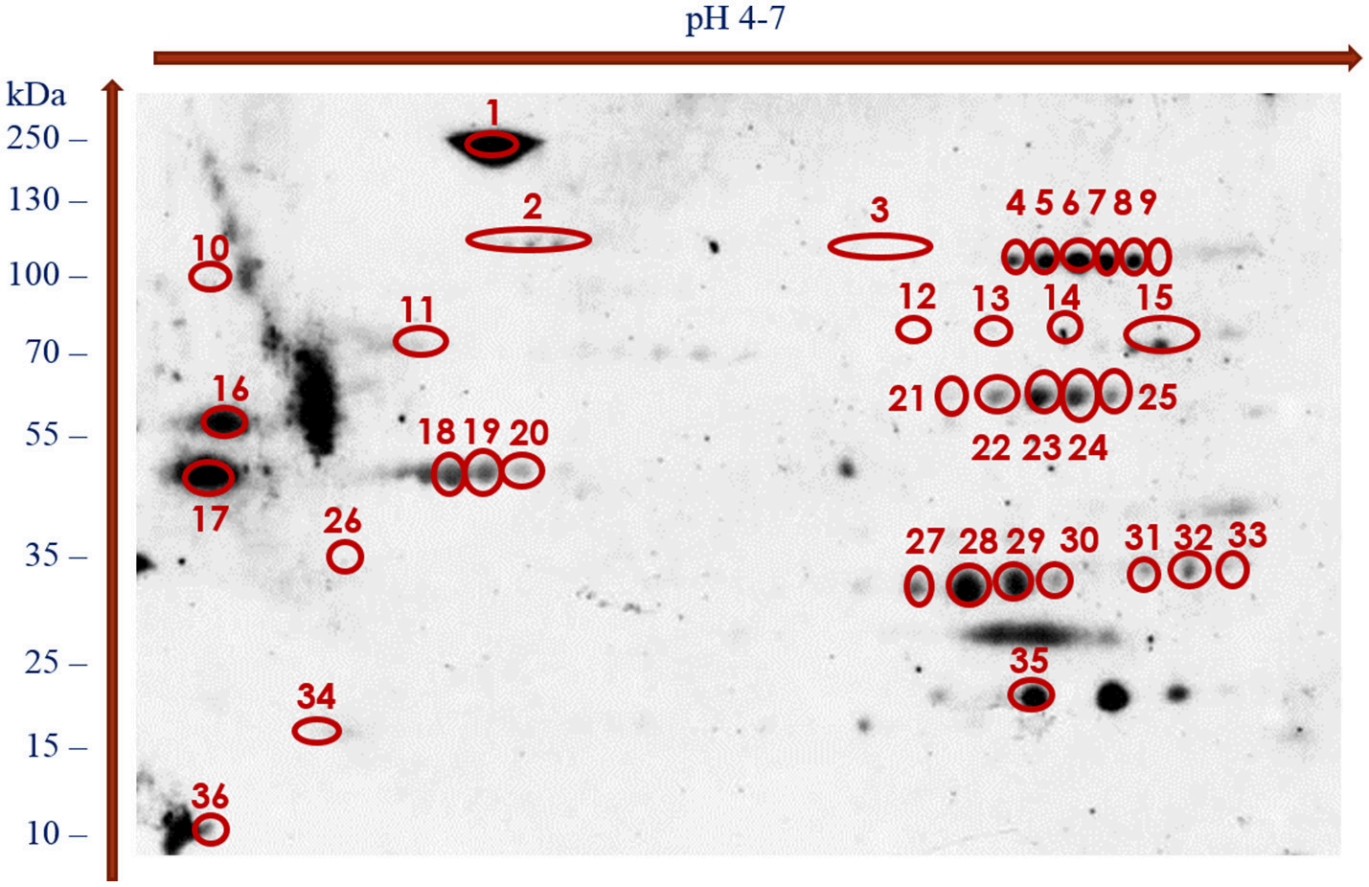
Recognition pattern of *H. diminuta* adult-stage immunoreactive protein spots by antibodies of *H. diminuta*-infected rats visualized using chemiluminescence.

With the use of the 2DE-immunoblotting we detected 36 spots as positively recognized by the *H. diminuta*-infected rat sera, whereas sera collected form uninfected rats were signal free (Figure 2 and Supplementary Figure 1). Potentially immunogenic proteins migrated predominantly with MWs between 35 and 250 kDa and pHs ranging from 4 to 5 and 6 to 7 (Figure 2). Several immunoreactive spots were also detected in the area between 10 and 35 kDa. The proteins were organized in eight groups of horizontally adjacent immunoreactive spots as shown in Figures 1, 2.

### LC-MS/MS identification of antigenic and surface proteins of *H. diminuta* adult stage

Thirty-six protein spots cross-reacting with the rat antisera were excised from the silver-stained replica 2DE-gel and subjected to in-gel tryptic digestion and LC-MS/MS analyses. As a result, 68 potentially antigenic proteins were identified and are listed in Table 1 and Supplementary File 1. As shown in Table 1 numerous proteins were identified from multiple spots; 38 of the identified proteins were present in more than one spot (Table 1 and Supplementary File 1). Similarly, the number of proteins identified per spot varied from spot to spot; the highest number of proteins were identified from the spot number 12 (with 11 proteins) (Supplementary File 2). Only one protein was present in spots 10 (spectrin beta chain) and 36 (myosin essential light chain). Proteins that were most frequently identified from multiple spots included the aldo-keto reductase family 1 (present in 8 spots) proteins, glutamate dehydrogenase (in 9 spots), glyceraldehyde-3-phosphate dehydrogenase—GAPDH (in 8 spots), enolase, phosphoenolpyruvate carboxykinase and pyruvate kinase—PYK (in 7 spots). Altogether, 30 proteins were identified from individual spots (Table 1).

**Table 1:** Alphabetical list of identified adult *Hymenolepis diminuta* antigenic proteins with spot numbers and recognition of potentially signaling/secretory proteins (antigenic proteins identified for the first time in the adult cestode are indicated in bold).

**Protein [Organism]**	**Spot number (Number of spots)**	SP*
**3-oxoacyl-acyl-carrier-protein reductase**^#^ **[*****Hm*****]**	35 (1)	N
Actin, cytoplasmic 2 [*Eg*]	1, 2, 11, 18, 19, 20 (6)	N
Actin, partial [*Dd*]	18, 19, 20, 26 (4)	N
Actin-1 [*Eg*]	19 (1)	N
Actin-5^#^, partial [*Dd*]	19 (1)	N
**Alanine aminotransferase 2**^#^ **[*****Hm*****]**	12, 21, 22 (3)	N
**Aldo-keto reductase family 1, member B4**^#^ **[*****Hm*****]**	22, 27, 28, 29, 30, 32, 33, 35 (8)	N
**Alpha-tubulin [*****Hd*****]**	11 (1)	N
**Annexin A8 [*****Eg*****]**	18, 25 (2)	N
**Aspartyl tRNA synthetase, cytoplasmic [*****Hm*****]**	12, 13 (2)	N
**Calpain A**^#^ **[*****Hm*****]**	11 (1)	N
**Calumenin-B [*****Eg*****]**	26 (1)	**Y**
**Capping protein (actin filament) muscle Z line [*****Hm*****]**	31 (1)	N
Cytosolic malate dehydrogenase^#^ [*Hm*]	27, 28, 29, 30, 31 (5)	N
**Deoxyhypusine hydroxylase:monooxygenase**^#^ **[*****Hm*****]**	18, 19 (2)	N
**Dnaj subfamily A [*****Hm*****]**	21 (1)	N
**Ef hand family protein [*****Hm*****]**	27 (1)	**Y**
**Elongation factor 2**^#^ **[*****Hm*****]**	15 (1)	N
Enolase^#^ [*Hm*]	4, 12, 21, 22, 23, 24, 25 (7)	N
**Estradiol 17 beta-dehydrogenase [*****Eg*****]**	27 (1)	N
**Eukaryotic initiation factor 4A [*****Hm*****]**	19, 33 (2)	N
**Filamin**^#^ **[*****Hm*****]**	1, 2, 11 (3)	N
**Fructose-1,6-bisphosphate aldolase**^#^ **[*****Hm*****]**	32, 33 (2)	N
**Fumarate hydratase class I**^#^ **[*****Hm*****]**	14, 15 (2)	N
**Glucose-6-phosphate isomerase [*****Eg*****]**	15, 31 (2)	N
**Glutamate dehydrogenase**^#^ **[*****Hm*****]**	6, 7, 12, 14, 21, 22, 23, 24, 25 (9)	N
**Glutamate dehydrogenase, mitochondrial**^#^ **[*****Hm*****]**	12, 23 (2)	N
**Glyceraldehyde-3-phosphate dehydrogenase**^#^ **[*****Hm*****]**	9, 15, 28, 29, 30, 31, 32, 33 (8)	N
**Glycogen phosphorylase**^#^ **[*****Hm*****]**	1 (1)	N
**GTP-binding nuclear protein Ran [*****Hm*****]**	35 (1)	N
Heat shock cognate protein [*Eg*]	2 (1)	N
Heat shock protein [*Eg*]	2, 11 (2)	N
Heat shock protein 60 [*Em*]	11 (1)	N
Heat shock protein 70^#^ [*Hm*]	2, 3 (2)	N
**Heterogeneous nuclear ribonucleoprotein 87F [*****Eg*****]**	31, 32, 33 (3)	N
**Hypothetical transcript [*****Hm*****]**	2, 12, 13 (3)	N
**Inosine-5- monophosphate dehydrogenase 2 [*****Hm*****]**	25 (1)	N
**Lactate dehydrogenase**^#^ **[*****Hm*****]**	28, 30 (2)	N
**Lamin [*****Hm*****]**	2, 3 (2)	N
**Leucyl aminopeptydase [*****Eg*****]**	13 (1)	N
**Major egg antigen [*****Hm*****]**	11 (1)	N
**Major vault protein**^#^ **[*****Hm*****]**	1 (1)	N
**Myosin essential light chain [*****Hm*****]**	36 (1)	N
**Myosin heavy chain**^#^ **[*****Hm*****]**	1, 2 (2)	N
**Myosin regulatory light chain [*****Eg*****]**	34 (1)	N
**NADP-dependent malic enzyme**^#^ **[*****Hm*****]**	12, 13, 14, 15 (4)	N
**Neuronal calcium sensor [*****Hm*****]**	12, 13 (2)	N
Paramyosin [*Hm*]	1, 2, 3, 4, 11 (4)	N
**Phosphoenolpyruvate carboxykinase**^#^ **[*****Hm*****]**	4, 5, 6, 7, 8, 9, 15 (7)	N
**Phosphoglucomutase**^#^ **[*****Hm*****]**	12, 14 (2)	N
**Pseudouridine metabolizing bifunctional protein [*****Hm*****]**	9 (1)	N
**Pyruvate kinase**^#^ **[*****Hm*****]**	6, 9, 12, 13, 14, 15 (7)	N
**Spectrin alpha actinin**^#^ **[*****Hm*****]**	18, 26 (2)	N
**Spectrin beta chain [*****Hm*****]**	10, 16, 20 (3)	N
Stress-70 protein [*Eg*]	2 (1)	N
**Subfamily T1A non peptidase [*****Hm*****]**	34 (1)	N
**Succinate dehydrogenase flavoprotein**^#^ **[*****Eg*****]**	3 (1)	N
**Succinyl coenzyme A ligase**^#^ **[*****Hm*****]**	19, 20 (2)	N
**T-complex protein 1 subunit delta [*****Hm*****]**	14, 15 (2)	N
**T-complex protein 1 subunit zeta [*****Hm*****]**	12, 13 (2)	N
**Transketolase**^#^ **[*****Hm*****]**	4, 5 (2)	N
Triosephosphate isomerase^#^ [*Hm*]	35 (1)	N
**Tropomyosin [*****Hm*****]**	16, 17 (2)	N
**Tropomyosin 2 high molecular weight [*****Mc*****]**	17 (1)	N
**Tubulin**^#^ **[*****Se*****]**	11 (1)	N
**Tubulin beta chain**^#^ **[*****Eg*****]**	11, 26 (2)	N
**Vacuolar H**+ **atpase v1 sector subunit A [*****Hm*****]**	2 (1)	N
**V-type proton atpase catalytic subunit A [*****Eg*****]**	2 (1)	N

SP - signal peptide;

* - the presence of secretory/signal proteins predicted with the use of SignalP 4.1 Server software;

Y - potentially secretory protein; N - negative search results;

# - protein recognized among surface proteins;

*Dd - Diphyllobothrium dendriticum; Eg - Echinococcus granulosus; Em - Echinococcus multilocularis; Hd - Hymenolepis diminuta; Hm - Hymenolepis microstoma; Mc - Mesocestoides corti; Se - Spirometra erinaceieuropaei*.

Figure 1 shows the cross-reactive protein spots numbered with 1, 5–8, 18–20, 22–23, 28–29, and 35. They were found to match with the following proteins: actin, aldo keto reductase family 1 proteins, enolase, filamin, glutamate dehydrogenase, paramyosin, myosin, malate dehydrogenase, phosphoenolpyruvate carboxykinase, succinyl coenzyme-A ligase, spectrin, triosephosphate isomerase (TPI), and 3 oxoacyl acyl carrier protein reductase. The MW and pH values of these potential antigens ranged between 55 and 250 kDa and 4 and 7, respectively.

Several of the potentially antigenic *H. diminuta* adult-stage proteins could be classified as enzymes with 9 different subclasses, structural proteins and heat shock proteins (HSPs) (Tables 1, 2). The oxidoreductases were found to be the most dominating enzyme group for the identified proteins (13 proteins) (Table 2).

**Table 2:** Enzymatic proteins identified by LCMS/MS in immunoreactive spots of the adult tapeworm *Hymenolepis diminuta*.

**Enzyme classes**	**Protein names**
*Fructose-bisphosphate aldolase*	Fructose-1,6-bisphosphate aldolase
*Hydrolases*	Calpain A
	Leucyl aminopeptidase
	Vacuolar H+ ATPase v1 sector subunit A
	V-type proton ATPase catalytic subunit A
*Interconverting aldoses and ketoses*	Glucose-6-phosphate isomerase
	Triosephosphate isomerase
*Isomerases*	Phosphoglucomutase
	Triosephosphate isomerase
*Ligase*	Aspartyl tRNA synthetase cytoplasmic
	Succinyl coenzyme A ligase
*Lyases*	Enolase
	Fumarate hydratase
	Phosphoenolpyruvate carboxykinase
*Oxidoreductases*	3-oxoacyl-[acyl-carrier-protein] reductase
	Aldo keto reductase family 1 member B4
	Cytosolic malate dehydrogenase
	Deoxyhypusine hydroxylase
	Estradiol 17 beta-dehydrogenase
	Glutamate dehydrogenase
	Glutamate dehydrogenase, mitochondrial
	Glyceraldehyde-3-phosphate dehydrogenase
	Inosine-5′-monophosphate dehydrogenase
	Lactate dehydrogenase
	NADP-dependent malic enzyme
	Succinate dehydrogenase flavoprotein
*Proteasome endopeptidase complex*	Proteasome subunit alpha type
*Transferases*	Alanine aminotransferase 2
	Pyruvate kinase
	Transketolase

Using non-gel based proteomic approach (in-solution tryptic digestion of proteins coupled with LC-MS/MS identification) we were able to identify 76 proteins from the surface of *H. diminuta* adult stage worms. All these proteins were identified in each replicate with at least three matching peptides (Supplementary File 3). Among these surface-associated proteins, enzymes involved in various catalytic activities were suggested to be the most abundant protein group. In addition, heat shock proteins with potential moonlighting functions and classical structural proteins including actins, myosins, and tubulins were also identified. Notably, 31 from these surface proteins were detected as potential antigens in 2DE immunoblotting (Table 1, proteins marked with hashtag), which indicates that several of these antigens are excreted out of the worm.

### Gene ontology (GO) of the potentially antigenic proteins of *H. diminuta* adult stage

**Figure 3:**
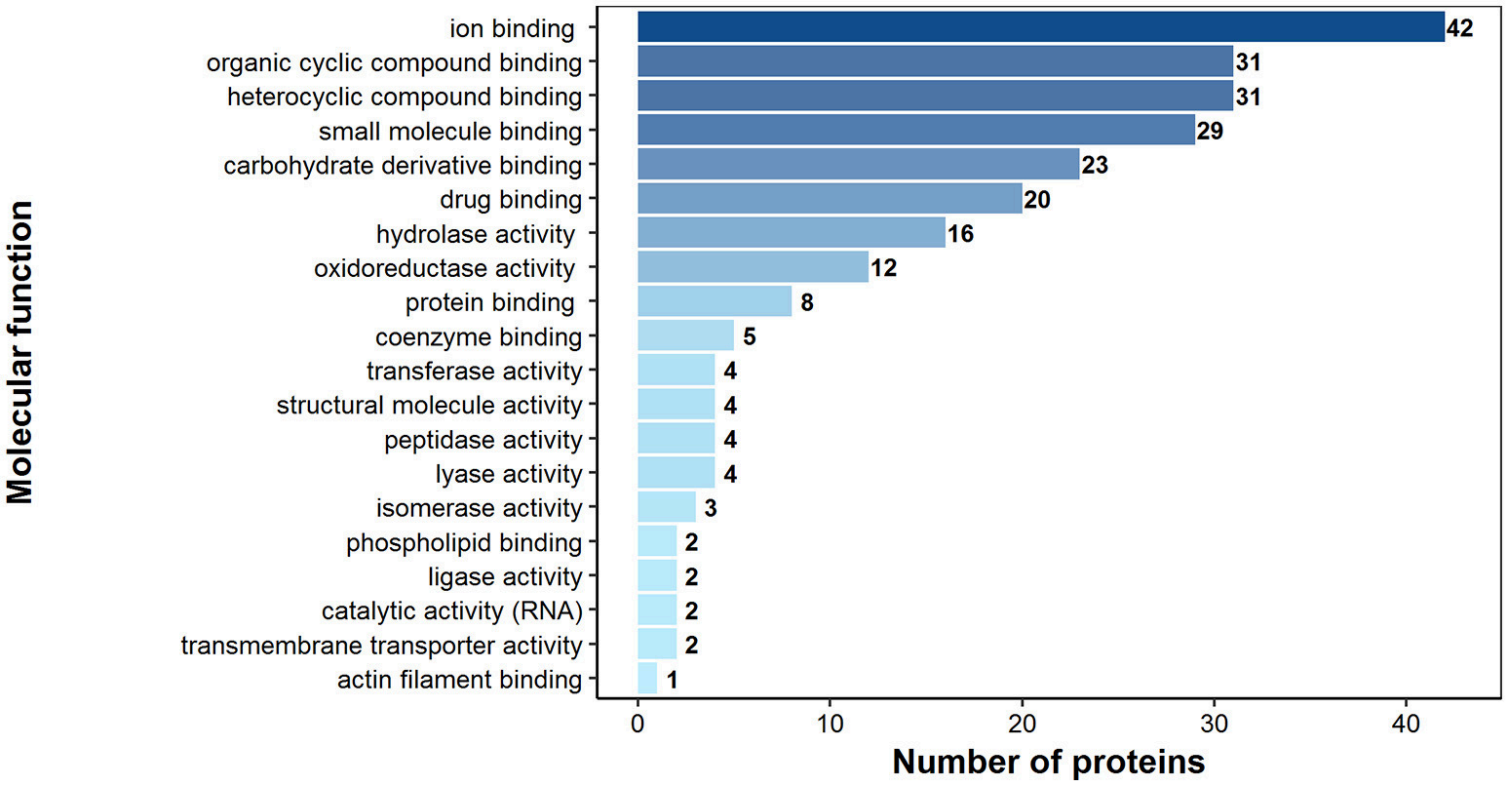
Identified *H. diminuta* adult stage antigenic proteins categorized by their molecular functions according to gene ontology (GO) information obtained from UniProtKB and QuickGO databases.

**Figure 4:**
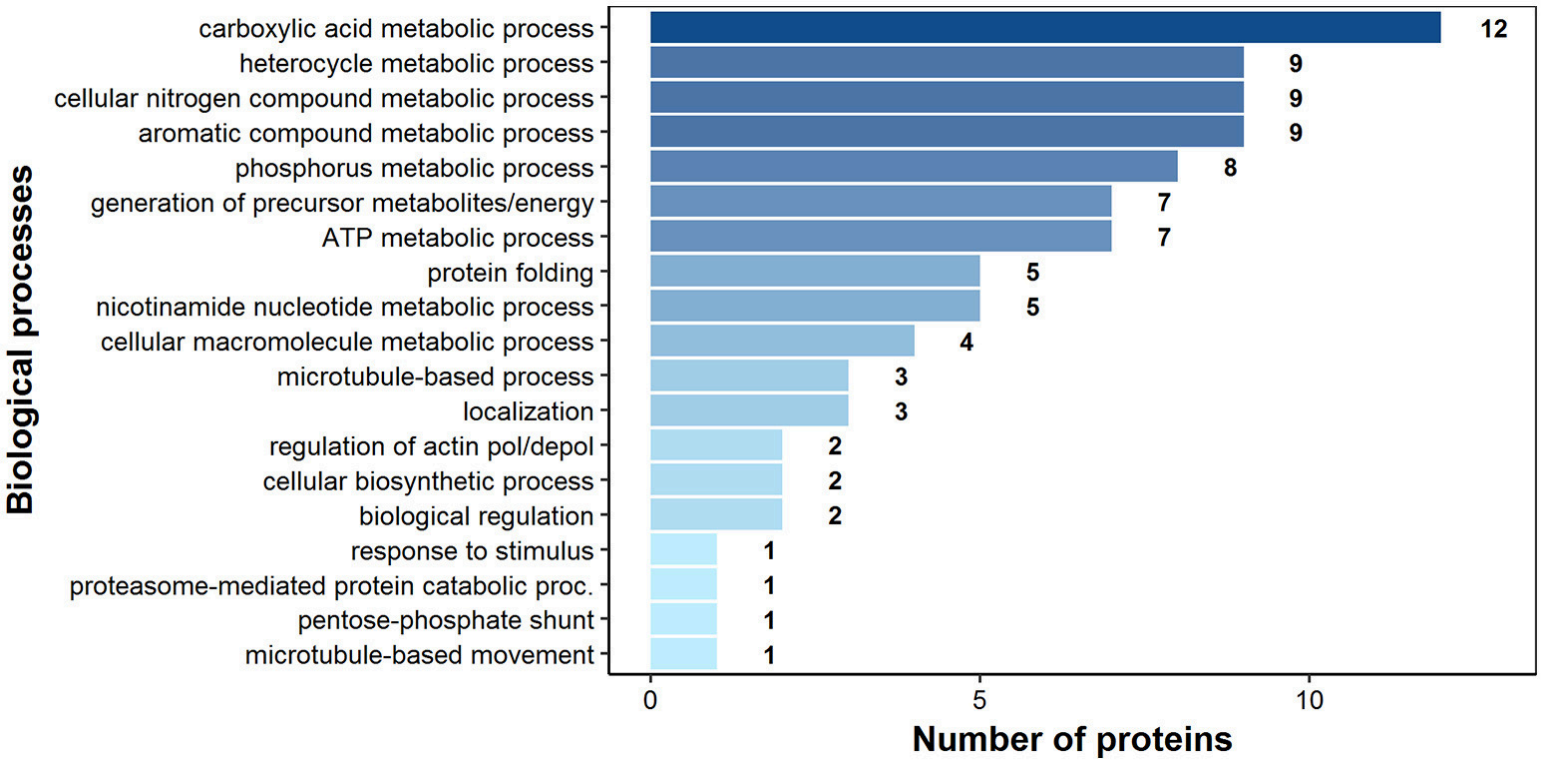
Identified *H. diminuta* adult-stage antigenic proteins categorized by their biological processes according to gene ontology (GO) information obtained from UniProtKB and QuickGO databases.

**Figure 5:**
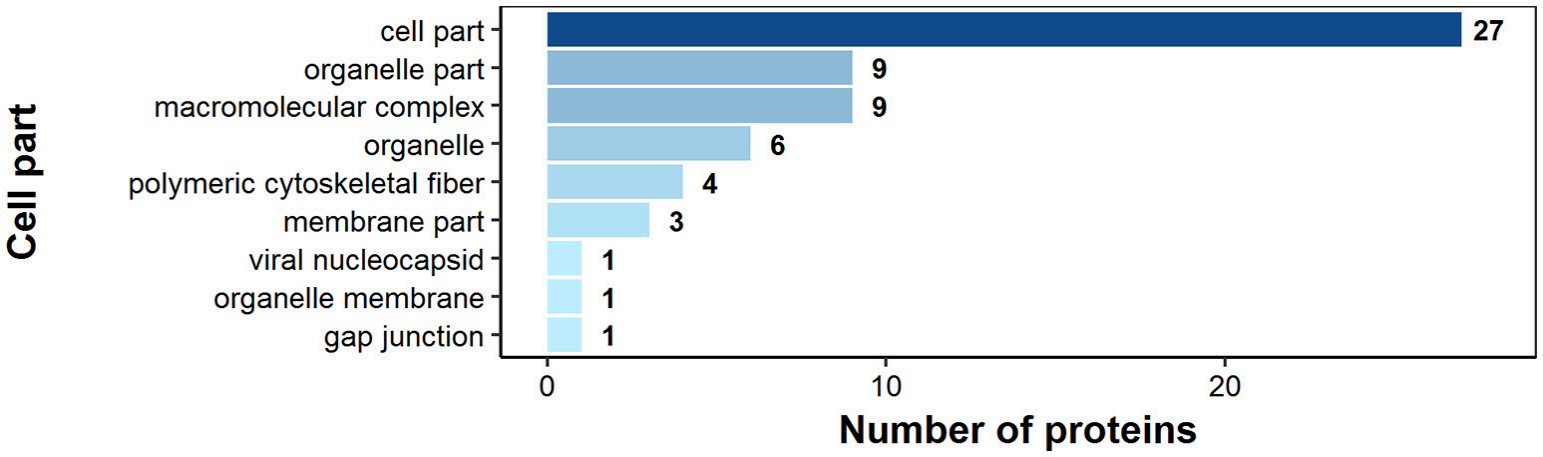
Identified *H. diminuta* adult-stage antigenic proteins categorized by their cellular component category according to gene ontology (GO) information obtained from UniProtKB and QuickGO databases.

According to bioinformatics predictions only 2 of the proteins were predicted to be secreted via the classical secretion pathway (calumenin-B, ef-hand family protein) (Table 1). Based on the GO annotation the identified proteins were classified into 3 different categories; molecular function (62 proteins), biological process (35 proteins), and as cellular components (30 proteins) (Figures 3–5). Twenty subcategories were assigned to molecular functions (Figure 3), with predominant groups related to binding, e.g., ion binding (42 proteins), organic cyclic compound binding (29), heterocyclic compound binding (29), and small molecule binding (27). Biological processes could be assigned to 35 proteins, most of them engaged with carboxylic acid metabolism (12), cellular nitrogen compound metabolism (9), aromatic compound metabolism (9), heterocycle (9) and phosphorus (8) metabolic processes (Figure 4). Figure 5 shows the distribution of the identified proteins according to their subcellular location; 30 proteins were associated with cellular structures (Figure 5), majority of them predicted to be localized in different cell (25) and organelle parts (9) or macromolecular complexes (9).

**Figure 6:**
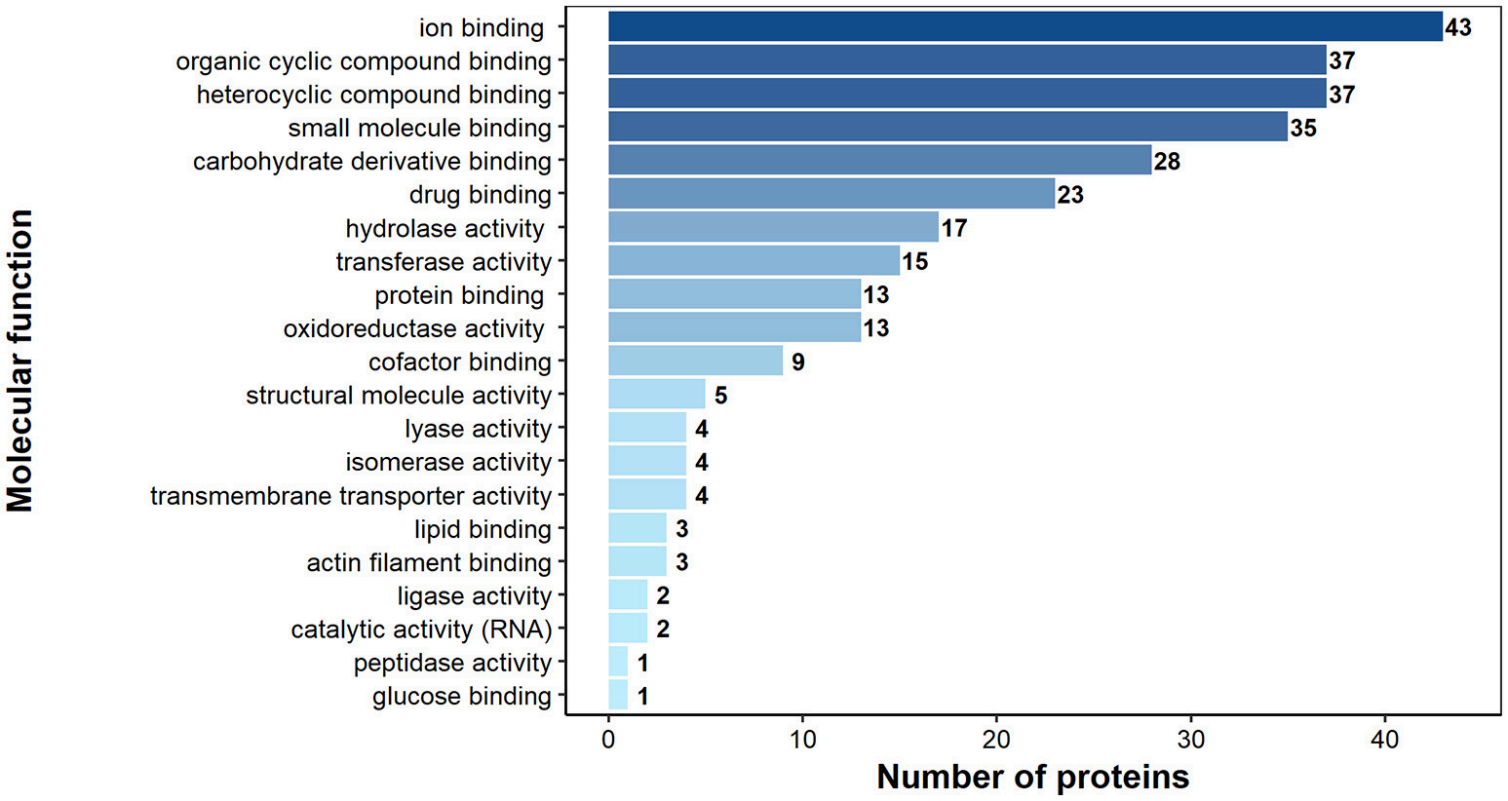
Identified *H. diminuta* adult stage surface proteins categorized by their molecular functions according to gene ontology (GO) information obtained from UniProtKB and QuickGO databases.

**Figure 7:**
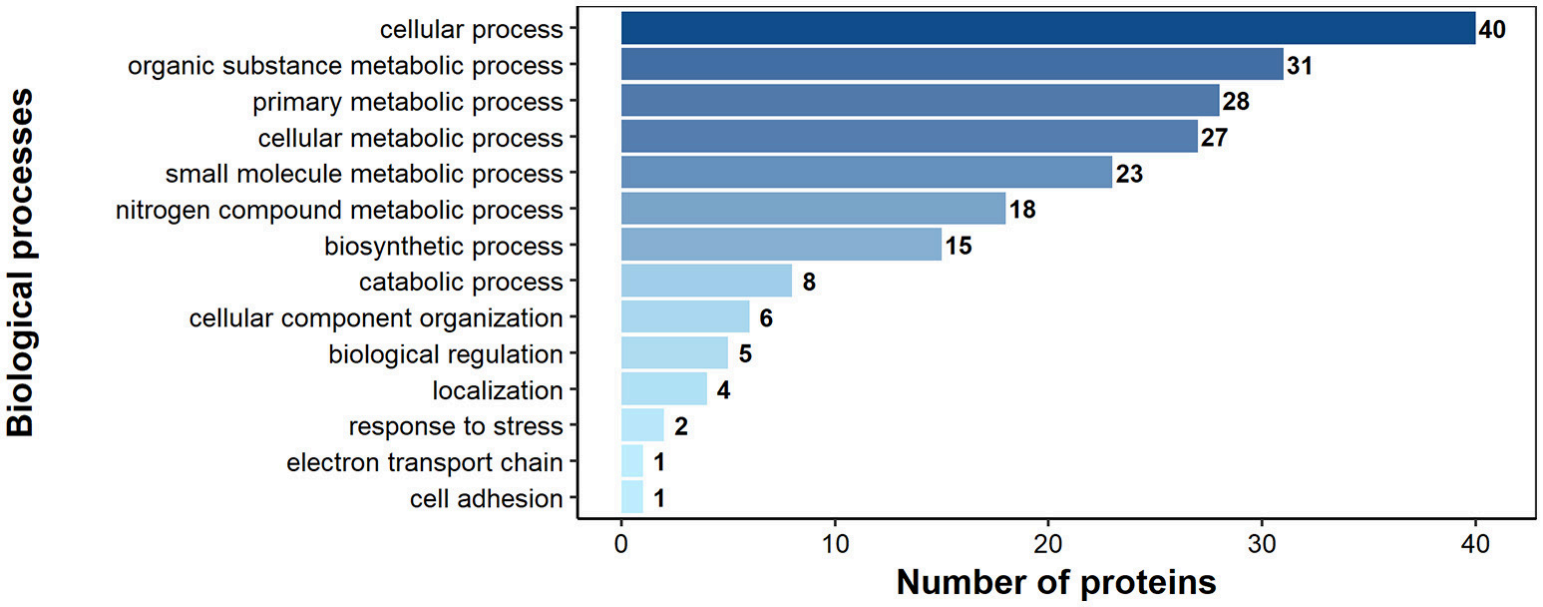
Identified *H. diminuta* adult stage surface proteins categorized by their biological processes according to gene ontology (GO) information obtained from UniProtKB and QuickGO databases.

**Figure 8:**
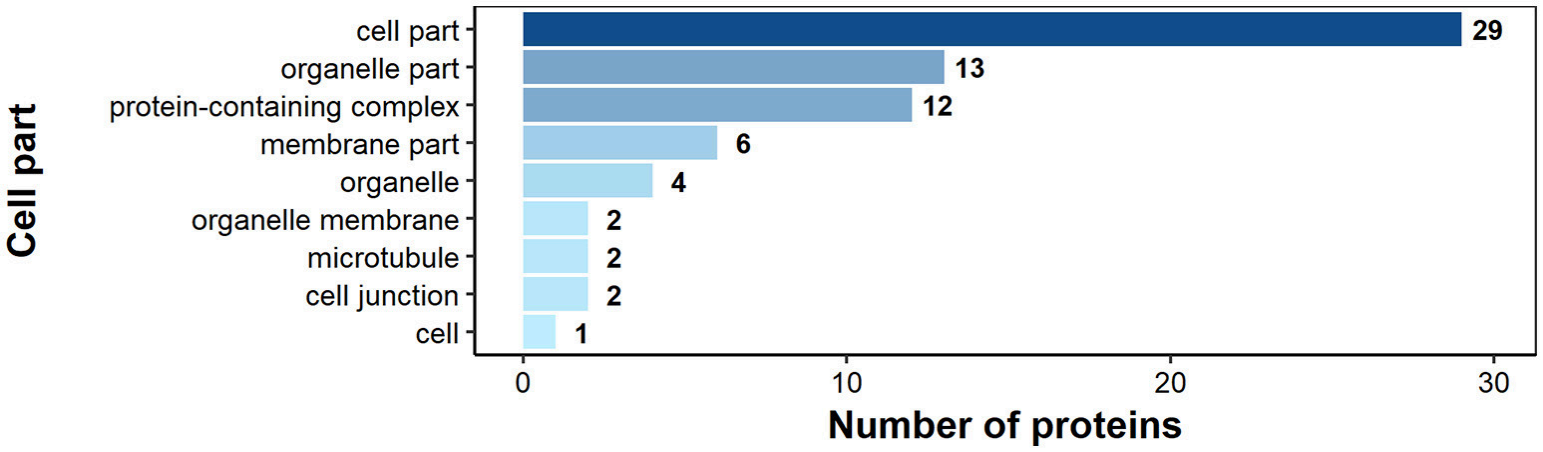
Identified *H. diminuta* adult stage surface proteins categorized by their cellular component category according to gene ontology (GO) information obtained from UniProtKB and QuickGO databases.

According to GO annotation 75 of the identified surface proteins were classified to molecular function, 48 to biological processes and 32 to associated cellular components (Figures 6–8). Among molecular functions the recognized proteins could be divided into 21 subcategories related mainly to molecule binding ion binding (43 proteins), organic (35) and heterocyclic compound binding (35), and small molecule binding (33) (Figure 6). The identified surface proteins associated with biological processes (Figure 7) are involved in cellular processes (40 proteins), organic substance (29) and primary metabolic (26) processes as well as in electron transport chain (25). Figure 8 illustrates the division of the identified surface proteins into different cellular compartments.

## Discussion

The present study shows that somatic proteins of the adult *H. diminuta* tapeworms exhibit immunogenicity in the rat host, and that the revealed immunoproteome could be used to propose new candidate proteins taking part in parasite–host interactions. Our previously published proteomic results of the *H. diminuta* (ESPs) show that the adult cestode immunogenic proteins were involved in stress response, various metabolic processes and structurally related functions (10). Some of these proteins have been considered as potential vaccine candidates and drug targets for treating e.g., schistosomiasis (37–39) and hydatidosis (40–42). Present study indicates slight differences between the protein profiles obtained from ESPs and somatic proteome. The ESPs, cross-reacting with the specific antibodies and not recognized in the somatic proteome, were predominantly identified as structural proteins (titin, myoferlin, gelsolin, and neurogenic locus notch protein), proteins associated with transport (basement membrane-specific heparan sulfate, phospholipid transporting ATPase, armadillo type fold) and ion-binding and/or ion channels (anoctamin, sarcoplasmic calcium-binding protein).

The identified immunoproteome of the adult *H. diminuta* tapeworm could be divided into two main groups: (i) structural proteins engaged in diverse parasite–host interactions and (ii) enzymes involved in key metabolic processes (NADP dependent malic enzyme, pyruvate kinase–PYK) or conferring moonlighting activity (43, 44) (e.g., triosephosphate isomerase—TPI, glycogen phosphorylase, L- lactate dehydrogenase–L-LDH, glyceraldehyde 3-phosphate dehydrogenase—GAPDH, glucose-6-phosphate isomerase-GPI, fructose 1,6-bisphosphate aldolase-FBA, inosine-5′-monophosphate dehydrogenase-IMP, succinate dehydrogenase, and deoxyhypusine hydroxylase) to the cells. Majority of these proteins are catalytic enzymes with a role in glycolysis, glyconeogenesis, tricarboxylic acid cycle, pyruvate fermentation or purine metabolism, which are cytosolic proteins predicted to be exported via non-classical secretion pathways and having adhesive moonlighting functions after entering the cell surface of an organism (43, 44). Since cestodes take the nutrients through the tegument and directly from the host, the direct contact of the host with the parasite-derived enzymes is highly probable. We may then hypothesize that the constant release of enzymes by the parasite not only influence host immunity but may also change the environment, and could explain the ability of the cestode to modify and alter the microbiome communities of the host intestine (14, 45–47). This is in consistent with the results of Kosik-Bogacka et al. who studied the impact of *H. diminuta* on transepithelial ion transport in intestines, and blood parameters of infected rats at different stages of the infection (24, 26). According to these observations cestodiasis reduced the transepithelial electrical potential and caused a leakage of tight junctions. Another effect of active hymenolepidosis was related to increased lipid peroxidation, changes in anti-oxidant enzyme activity and altered glutathion level in the infected rat gastrointestinal tract (25). This suggests decreased efficiency of intestinal protection against oxidative stress induced by the presence of the parasite. All of the aforementioned processes and the presence of parasite derived molecules may change the intestinal environment and provoke changes in microbiome composition.

On the other hand, as suggested by Kosik-Bogacka (24–26) imbalance between oxidant and anti-oxidant processes can play a major role in pathology associated with hymenolepidosis, including its proinflamatory role associated with the expression and activation of cyclooxygenases in the rat gastrointestinal tract (31). Pathomechanisms observed during infection with *H. diminuta* may be connected with the changes in the expression of toll-like receptors (TLR) important for pathogen recognition. Kosik-Bogacka et al. (28, 29), studying the expression of TLR in *H. diminuta* infected rats, observed increased TLR2 and TLR4 expression compared to the control group, especially in the small intestine. Similarly, the TLR3 snf TLT9 expression was higher in infected rats (30). These analyses confirmed the role of TLR in hymenolepidosis and suggest that *H. diminuta* releases antigens stimulating immune response, which results in adaptation of the host organism to parasite derived molecules (32). Our previous research demonstrated the presence of PYK and GAPDH among the ESP of adult *H. diminuta* (10) and here we confirmed their immunogenic potential. In addition to glycolytic enzymes (PYK and GAPDH), also these taking part in fatty enzyme metabolism are considered important drug targets. Similarly to 3-oxoacyl-ACP reductase, an important candidate in antischistosomal combat (48). As the above mentioned enzymatic proteins have not been identified as immunogenic in the adult *E. granulosus* (9), our study reports their possible antigenicity in an adult tapeworms for the first time. This suggests that similarly to metacestodes, adult tapeworm enzymes (moonlighters) take part in parasite-host interplay. Some of these metabolic enzymes may be derived from the tegument by shedding of glycocalyx (present paper) or are released with secretory vesicles as described by Ancarola et al. (49) for metacestodes.

Our findings suggest that the non-classical protein export, possibly involving protein moonlighters, could contribute to increased viability and interaction with the host. Intriguingly, a recent study reported that this interplay may be mediated by the involvement of extracellular vesicles (EVs) to export cytosolic proteins in a protected and concentrated manner, as proved for metacestodes (49). Similarly, EVs released by hexacanth larvae were proved to be a source of *Taenia ovis* vaccine antigens (50). Since our immunoproteomic approach shows the presence of certain cytosolic and structural proteins as possible antigens, we suppose that the mechanism of their trafficking may have involved EVs. These include for instance antigens considered as vaccine candidates such as calpain (51, 52) and the major egg antigen p40 (mp40) (53–55).

Structural and enzymatic proteins are the most typical immunodominant antigens represented by the somatic proteome of the adult stage of *H. diminuta*. Among cestodes, antigenicity of certain structural proteins was first described in studies focusing on the metacestode stages (40–42, 56–61). Our recent study on antigenic proteins of *H. diminuta* cysticercoid metacestodes (57) supported these findings and confirmed the immunogenic importance of structural proteins, in host's immune response to infection. This indicated that structural proteins, for example beta-tubulin, should be considered as vaccine candidates and/or drug targets against cestode metacestode and adult stages. Another interesting example of structural protein is paramyosin identified at the helminths' surface or among the secreted proteins. This structural protein is believed to function as a multifunctional modulator of the host immune response (62, 63), and together with actin is involved in tegumental repair and considered to represent an important vaccine target molecule. The localization of this protein in the tegument of the parasite is the likely basis for resistance observed in mice immunized with paramyosin (64). Interestingly, it has been speculated that paramyosin protects invading helminths from the immune attack by “decoy” binding proteins of the complement pathway (62). The presence of paramyosin has also been identified as a potential antigen in adult tapeworms (51). Antigenicity of paramyosin was further confirmed by Wang et al. (9), as this protein was one of the major antigens recognized by the antisera from dogs infected with adult *Echinococcus granulosus*.

Immunoproteomic analysis of adult *E. granulosus* has revealed the presence of 12 and 8 potentially antigenic proteins associated, respectively with the somatic proteome and the secretome of this organism (9). Only 7 potentially immunogenic proteins were commonly identified from *E. granulosus* and by us in *H. diminuta* adult worms. These included actin, paramyosin and several moonlighters such as enolase (ENO), malate dehydrogenase (MDH), TPI as well as the stress-related HSP60 and HSP70 proteins. Two other proteins, namely, calreticulin and superoxide dismutase (SOD) were previously identified in *H. diminuta* cysticercoids (11), but they have not been recognized as immunogenic (57). The revealed immunoproteome of the *H. diminuta* indicated potential vaccine candidates against echinococcosis, such as ENO, calpain, and GAPDH and the stress proteins HSP60 and HSP70 (51). Heat shock proteins (HSPs) are known key players in processes associated with development, differentiation, survival, aging, and death. While the antigenic potential of HSPs was shown in previous studies on cestode metacestode stages (56, 57, 65), their immunogenicity in the adult tapeworms has been confirmed only in *E. granulosus* (9), in the present and in our previous studies on *H. diminuta* ESP (10). For as much as HSPs are considered as potential vaccine target proteins (66), their presence throughout the cestode life cycle suggests their importance in cestode biology and survival in the host. The balanced interplay between structural and stress molecules is probably one of the survival factors adopted by parasites during coevolution with their hosts (11). Comparative proteomic analyses of adult and metacestode stages suggest stage-specific mechanisms engaged in the parasite's survival at different life-cycle stages (11, 67). In *H. diminuta*, proteins with antigenic potential, common for these two stages are structural proteins (actins, annexin, lamin, myosin, paramyosin, tubulin), enzymes (calpain, NADP dependent malic enzyme, phosphoenolpyruvate carboxykinase, succinyl co-A), HSPs, and major egg antigen (57). Differences in the expression of immunogenic proteins observed in these two distinct stages are associated predominantly with enzymes and may reflect variability in metabolic activity and stage-specific survival strategies.

There has been interest in targeting metabolic enzymes in the treatment of infectious diseases (68). Fumarate hydratase, involved in canalization of the stereospecific reversible hydration of fumarate to l-malate during the citric acid cycle, was one of the enzymes we have previously identified as one of the immunogens characteristic for the adult *H. diminuta* (11). It has been shown that this enzyme takes part in dismutation of malate in the nematode mitochondria (69), thereby making this enzyme one of the promising targets for designing efficient anthelminthic drugs. The present study also suggests that non-classically exported cytoplasmic proteins, i.e., moonlighters (e.g., enzymes, structural proteins, and HSPs), form another group of proteins playing important roles in host–parasite interactions. One of these moonlighters may be TPI that has also been identified as one of the somatic and surface proteins in *H. diminuta* (9) and in the present study. This enzyme is involved in glycolysis and has been proposed to represent a potential drug target and vaccine candidate to treat schistosomiasis (70).

The potential role of parasite antigens in contributing to increased viability via their immunomodulatory and anti-inflammatory activity has resulted in the concept of helminth-derived molecules as a source of immunomodulatory agents (32, 71, 72). The immunogenic proteins in the crude extract and among the identified surface proteins of *H. diminuta* adult worms may show potential in search of new drug targets and diagnostic methods, since the helminth-derived molecules are considered as potential therapeutics of autoimmune and inflammatory diseases (32–34, 72–74). Recent reports indicated that adult *H. diminuta* tapeworms can effectively modulate the host immune system (15, 16, 20, 47, 75, 76), and that *H. diminuta-*derived molecules can be used to control inflammation (20, 75, 77–79). The results here indicate that *H. diminuta* somatic proteins have been exposed to the host immune system as antigens. However, to uncover full immunomodulatory potential of the identified *H. diminuta* proteins, further studies and finally *in vivo* experiments are needed.

## Conclusions

The somatic proteome and surfaceome of the adult *H. diminuta* with the use of 2DE immunoblotting and LC-MS/MS identification is reported here. The present study proposed a number of new immunogenic proteins involved in key metabolic processes and with likely roles in mediating parasite–host interactions. The identified immunodominant antigens, classified as proteins having structural and enzymatic functions, suggest contributory role of these molecules in mediating host–parasite interaction and during the adult cestode infection. Here we point to enzymes, structural and heat-shock proteins as potential mediators in the interactions between the parasite and the host. Most of these molecules are predicted to trafficate by non-classical ways by motifs or signals that remain to be uncovered. Thus, the present study shed new light on the complexity of the parasite-host interplay during cestodiasis, and highlights the importance of non-classical protein export (e.g., EVs and protein moonlighters) in modulating the parasite–host interaction. This study also provides valuable data not only for understanding the adult cestode biology but also for searching new targets for diagnostic and drug innovations.

## Author contributions

DM supervised the work and all its proteomic features and drafted the manuscript. KS and AN participated in the planning of the study. AN, DM, JB, and KS conceived and designed the experiments. AS, JB, and DM conceived and performed the immunological study with rats. KS supported AS and DM in mass spectrometry data analyses. AN, AZ-D, DBC, JB, KB, KS, and RS participated in data analyses and final editing of the manuscript. All authors were actively involved in preparing the manuscript with the responsible author. All authors read and approved the final version of the manuscript.

### Conflict of interest statement

The authors declare that the research was conducted in the absence of any commercial or financial relationships that could be construed as a potential conflict of interest.

## Supplementary material

Supplementary Figure 1 Negative control showing Western blot recognition pattern of *H. diminuta* adult-stage proteins with sera collected from *H. diminuta*-uninfected rats and visualized using chemiluminescence.

Supplementary File 1 Results of the LC-MS/MS analysis of immunoreactive protein spots of the adult tapeworm somatic proteome recognized by sera from *Hymenolepis diminuta* infected rats.

Supplementary File 2 *Hymenolepis diminuta* adult stage proteins identified from immunoreactive spots selected for LC-MS/MS analysis.

Supplementary File 3 Results of the LC-MS/MS analysis of collected surface proteins of the adult tapeworm *Hymenolepis diminuta*.
