# Supplementary figures and images for: Immunoproteomics and Surfaceomics of the Adult Tapeworm Hymenolepis diminuta
Source: Front Immunol. 2018 Nov 12;9:2487. doi: 10.3389/fimmu.2018.02487 (PMC6240649; doi:10.3389/fimmu.2018.02487)

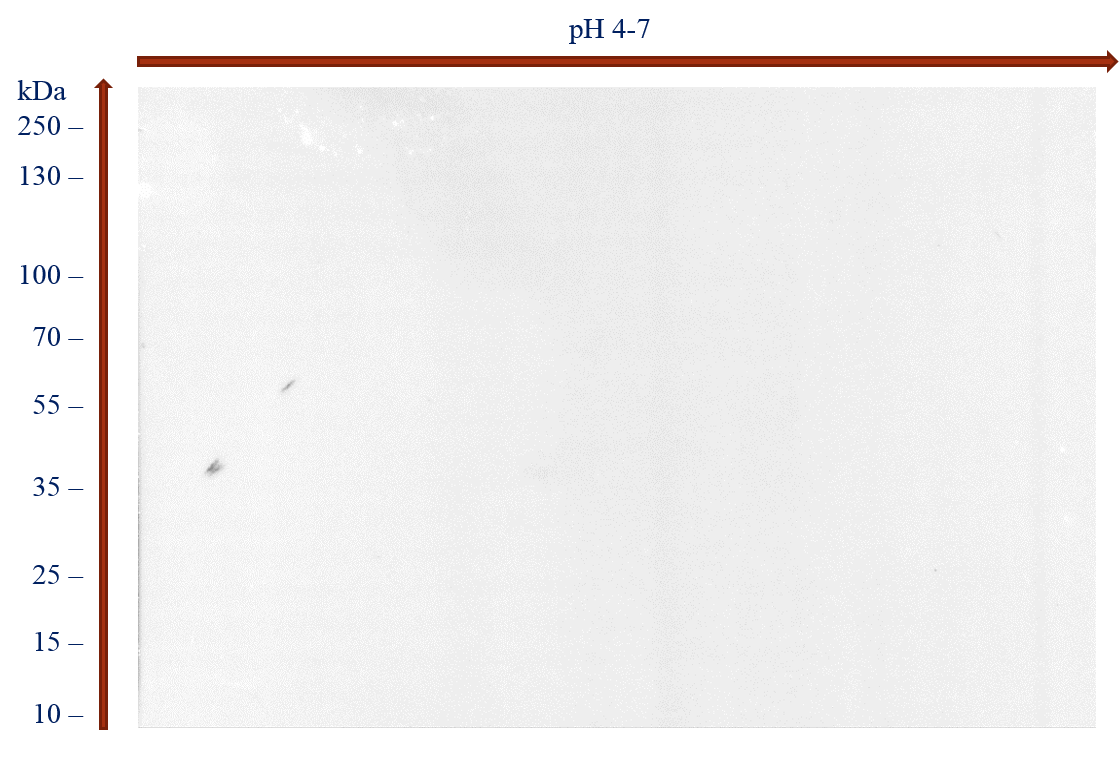

Supplement: Supplementary Figure 1 — Negative control showing Western blot recognition pattern of H. diminuta adult-stage proteins with sera collected from H. diminuta-uninfected rats and visualized using chemiluminescence. [file Image_1.TIF]
