# Supplementary material for: Immunoproteomics and Surfaceomics of the Adult Tapeworm Hymenolepis diminuta
Source: Front Immunol. 2018 Nov 12;9:2487. doi: 10.3389/fimmu.2018.02487 (PMC6240649; doi:10.3389/fimmu.2018.02487)
Supplement: Supplementary File 1 — Results of the LC-MS/MS analysis of immunoreactive protein spots of the adult tapeworm somatic proteome recognized by sera from Hymenolepis diminuta infected rats. [file Table_1.DOCX]

**Supplementary File 1.** Results of the LC-MS/MS analysis of immunoreactive protein spots of the adult tapeworm somatic proteome recognized by sera from *Hymenolepis diminuta* infected rats.

| **Database** | **Accession** | **Score** | **Mass** | **Matches** | **Sequences** | **emPAI** | **% cover** | **Protein [Organism]** |
| --- | --- | --- | --- | --- | --- | --- | --- | --- |
| NCBI | CDS30447.1 | 1346 | 27863 | 26 | 6 | 1.47 | 26 | 3-oxoacyl-acyl-carrier-protein reductase [*Hm*] |
| NCBI | EUB56832.1 | 5917 | 42176 | 150 | 18 | 7.91 | 42 | Actin, cytoplasmic 2 [*Eg*] |
| NCBI | AAA21482.1 | 6526 | 41700 | 166 | 18 | 10.46 | 43 | actin, partial [*Dd*] |
| NCBI | P35432.1 | 1431 | 42143 | 37 | 9 | 1.88 | 24 | Actin-1 [*Eg*] |
| NCBI | P53458.1 | 5508 | 41442 | 143 | 16 | 6.37 | 25 | Actin-5, partial [*Dd*] |
| NCBI | CDS31970.1 | 329 | 52764 | 7 | 7 | 0.53 | 13 | alanine aminotransferase 2 [*Hm*] |
| NCBI | CDS26971.1 | 2487 | 36405 | 101 | 11 | 2.39 | 20 | aldo-keto reductase family 1, member B4 [*Hm*] |
| NCBI | AAL84895.1 | 203 | 51217 | 5 | 5 | 0.37 | 10 | alpha-tubulin [*Hd*] |
| NCBI | EUB64725.1 | 232 | 46978 | 4 | 4 | 0.31 | 8 | Annexin A8 [*Eg*] |
| NCBI | CDS30263.1 | 344 | 58980 | 7 | 7 | 0.46 | 13 | aspartyl tRNA synthetase, cytoplasmic [*Hm*] |
| NCBI | CDS34356.1 | 129 | 88988 | 4 | 4 | 0.16 | 3 | calpain A [*Hm*] |
| NCBI | EUB65028.1 | 634 | 36814 | 13 | 5 | 0.83 | 13 | Calumenin-B [*Eg*] |
| NCBI | CDS30894.1 | 257 | 33925 | 4 | 4 | 0.45 | 13 | capping protein (actin filament) muscle Z line [*Hm*] |
| NCBI | CDS29929.1 | 439 | 37321 | 11 | 6 | 0.81 | 18 | cytosolic malate dehydrogenase [*Hm*] |
| NCBI | CDS31349.1 | 233 | 39225 | 6 | 4 | 0.38 | 10 | deoxyhypusine hydroxylase:monooxygenase [*Hm*] |
| NCBI | CDS28557.1 | 198 | 45252 | 4 | 4 | 0.33 | 10 | dnaJ subfamily A [*Hm*] |
| NCBI | CDS30131.1 | 495 | 37252 | 18 | 9 | 1.78 | 18 | ef hand family protein [*Hm*] |
| NCBI | CDS32105.1 | 141 | 95061 | 4 | 4 | 0.15 | 3 | elongation factor 2 [*Hm*] |
| NCBI | AAC47639.1 | 1432 | 47223 | 33 | 16 | 3.73 | 19 | enolase [*Hm*] |
| NCBI | EUB55283.1 | 204 | 36221 | 6 | 3 | 0.42 | 5 | Estradiol 17 beta-dehydrogenase [*Eg*] |
| NCBI | CDS27603.1 | 195 | 44771 | 4 | 4 | 0.33 | 10 | eukaryotic initiation factor 4A [*Hm*] |
| NCBI | CDS33789.1 | 411 | 274858 | 7 | 7 | 0.09 | 3 | filamin [*Hm*] |
| NCBI | CDS26447.1 | 255 | 39942 | 6 | 5 | 0.61 | 14 | fructose 1,6 bisphosphate aldolase [*Hm*] |
| NCBI | CDS31600.1 | 423 | 62454 | 9 | 8 | 0.59 | 16 | fumarate hydratase class I [*Hm*] |
| NCBI | AAX73170.1 | 218 | 61549 | 4 | 3 | 0.23 | 5 | Glucose-6-phosphate isomerase [*Eg*] |
| NCBI | CDS29594.1 | 2052 | 58437 | 65 | 7 | 0.73 | 9 | glutamate dehydrogenase [*Hm*] |
| NCBI | CDS25323.1 | 454 | 55360 | 21 | 3 | 0.26 | 3 | Glutamate dehydrogenase, mitochondrial [*Hm*] |
| NCBI | CDS31614.1 | 1026 | 36804 | 28 | 7 | 1.17 | 20 | Glyceraldehyde-3-phosphate dehydrogenase [*Hm*] |
| NCBI | CDS27901.2 | 151 | 97910 | 5 | 5 | 0.18 | 7 | glycogen phosphorylase [*Hm*] |
| NCBI | CDS33870.1 | 248 | 24354 | 5 | 4 | 0.67 | 5 | GTP-binding nuclear protein Ran [*Hm*] |
| NCBI | EUB56318.1 | 919 | 71324 | 23 | 16 | 1.46 | 21 | Heat shock cognate protein [*Eg*] |
| NCBI | EUB62115.1 | 987 | 64625 | 19 | 14 | 1.21 | 22 | heat shock protein [*Eg*] |
| NCBI | CDS35950.1 | 994 | 60984 | 17 | 13 | 1.09 | 22 | heat shock protein 60 [*Em*] |
| NCBI | CDS28178.1 | 1230 | 70972 | 31 | 19 | 2.1 | 28 | heat shock protein 70 [*Hm*] |
| NCBI | EUB64805.1 | 250 | 32030 | 6 | 5 | 0.81 | 17 | Heterogeneous nuclear ribonucleoprotein 87F [*Eg*] |
| NCBI | CDS32495.2 | 281 | 113235 | 7 | 7 | 0.22 | N/A | hypothetical transcript [*Hm*] |
| NCBI | CDS32058.1 | 185 | 55082 | 4 | 4 | 0.26 | 7 | Inosine-5'-monophosphate dehydrogenase 2 [*Hm*] |
| NCBI | CDS26883.1 | 117 | 36385 | 5 | 4 | 0.42 | 6 | lactate dehydrogenase a [*Hm*] |
| NCBI | CDS25470.1 | 323 | 49810 | 10 | 10 | 0.9 | 19 | lamin [*Hm*] |
| NCBI | EUB62177.1 | 211 | 56176 | 5 | 4 | 0.33 | 7 | leucyl aminopeptidase [*Eg*] |
| NCBI | CDS33705.1 | 288 | 52831 | 6 | 5 | 0.35 | 10 | major egg antigen [*Hm*] |
| NCBI | CDS29747.1 | 250 | 97866 | 7 | 6 | 0.26 | 5 | major vault protein [*Hm*] |
| NCBI | CDS33375.1 | 1712 | 20437 | 55 | 4 | 1.49 | 23 | myosin essential light chain [*Hm*] |
| NCBI | CDS33163.1 | 924 | 224303 | 20 | 20 | 0.33 | 10 | myosin heavy chain [*Hm*] |
| NCBI | EUB59497.1 | 385 | 21432 | 12 | 7 | 2.2 | 35 | Myosin regulatory light chain [*Eg*] |
| NCBI | CDS31690.2 | 277 | 65618 | 8 | 8 | 0.48 | 10 | NADP-dependent malic enzyme [*Hm*] |
| NCBI | CDS29101.2 | 180 | 56386 | 6 | 5 | 0.41 | 6 | neuronal calcium sensor [*Hm*] |
| NCBI | CDS32748.1 | 8749 | 99179 | 194 | 52 | 10.79 | 32 | paramyosin [*Hm*] |
| NCBI | CDS27807.1 | 709 | 71158 | 14 | 10 | 0.8 | 14 | phosphoenolpyruvate carboxykinase [*Hm*] |
| NCBI | CDS26093.1 | 185 | 127353 | 4 | 4 | 0.11 | 3 | phosphoglucomutase [*Hm*] |
| NCBI | CDS29349.1 | 302 | 75507 | 6 | 5 | 0.29 | 7 | pseudouridine metabolizing bifunctional protein [*Hm*] |
| NCBI | CDS25961.1 | 612 | 114130 | 15 | 11 | 0.4 | 11 | pyruvate kinase [*Hm*] |
| NCBI | CDS29622.1 | 251 | 282888 | 6 | 6 | 0.07 | 2 | Spectrin alpha actinin [*Hm*] |
| NCBI | CDS28968.2 | 314 | 272698 | 6 | 6 | 0.07 | 2 | spectrin beta chain [*Hm*] |
| NCBI | EUB60044.1 | 822 | 78208 | 18 | 15 | 1.01 | 19 | Stress-70 protein [*Eg*] |
| NCBI | CDS31842.1 | 528 | 26634 | 9 | 7 | 1.89 | 32 | subfamily T1A non peptidase [*Hm*] |
| NCBI | EUB62925.1 | 657 | 71838 | 12 | 9 | 0.71 | 15 | Succinate dehydrogenase flavoprotein [*Eg*] |
| NCBI | CDS31153.1 | 249 | 47516 | 7 | 4 | 0.31 | 7 | succinyl coenzyme A ligase [*Hm*] |
| NCBI | CDS26996.1 | 473 | 58251 | 10 | 10 | 0.73 | 19 | T-complex protein 1 subunit delta [*Hm*] |
| NCBI | CDS29679.2 | 383 | 60491 | 7 | 7 | 0,45 | 9 | T-complex protein 1 subunit zeta [*Hm*] |
| NCBI | CDS28549.1 | 137 | 68433 | 4 | 4 | 0.21 | 4 | transketolase [*Hm*] |
| NCBI | CDS29962.1 | 923 | 27872 | 26 | 8 | 2.09 | 28 | triosephosphate isomerase [*Hm*] |
| NCBI | CDS32881.1 | 616 | 45208 | 15 | 8 | 1.02 | 17 | tropomyosin [*Hm*] |
| NCBI | ADQ26722.1 | 852 | 31588 | 20 | 11 | 2.66 | 33 | Tropomyosin 2 high molecular weight [*Mc*] |
| NCBI | AFX72996.1 | 181 | 50838 | 5 | 5 | 51 | 10 | tubulin [*Se*] |
| NCBI | EUB57572.1 | 263 | 50258 | 5 | 4 | 0.29 | 10 | Tubulin beta chain [*Eg*] |
| NCBI | CDS25629.1 | 486 | 128305 | 10 | 10 | 0.29 | 8 | vacuolar H+ ATPase v1 sector subunit A [*Hm*] |
| NCBI | EUB60320.1 | 395 | 69149 | 7 | 7 | 0.38 | 12 | V-type proton ATPase catalytic subunit A [*Eg*] |

*Dd* – *Diphyllobothrium* *dendriticum*; *Eg* – *Echinococcus* *granulosus*; *Em* – *Echinococcus* *multilocularis*; *Hd* – *Hymenolepis* *diminuta*; *Hm* – *Hymenolepis* *microstoma*; *Mc* – *Mesocestoides* *corti*; *Se* – *Spirometra* *erinaceieuropaei*;
