# Supplementary material for: Immunoproteomics and Surfaceomics of the Adult Tapeworm Hymenolepis diminuta
Source: Front Immunol. 2018 Nov 12;9:2487. doi: 10.3389/fimmu.2018.02487 (PMC6240649; doi:10.3389/fimmu.2018.02487)
Supplement: Supplementary File 3 — Results of the LC-MS/MS analysis of collected surface proteins of the adult tapeworm Hymenolepis diminuta. [file Table_3.DOCX]

**Supplementary File 3.** Results of the LC-MS/MS analysis of collected surface proteins of the adult tapeworm *Hymenolepis diminuta*.

| **Database** | **Accession** | **Score** | **Mass** | **Matches** | **Sequences** | **emPAI** | **% cover** | **Protein [Organism]** |
| --- | --- | --- | --- | --- | --- | --- | --- | --- |
| NCBIprot | CDS28921.1 | 657 | 29005 | 10 | 5 | 1.06 | 28 | 14-3-3 protein epsilon [*Hm*] |
| NCBIprot | CDS30447.1 | 1798 | 27819 | 46 | 4 | 0.82 | 20 | 3 oxoacyl acyl carrier protein reductase [*Hm*] |
| NCBIprot | CDS33697.1 | 532 | 89854 | 24 | 8 | 0.46 | 11 | 6 phosphofructokinase [*Hm*] |
| NCBIprot | CDS23123.1 | 402 | 56240 | 11 | 3 | 0.25 | 14 | Acetyl coenzyme A hydrolase transferase [*Eg*] |
| NCBIprot | CDS21485.1 | 5660 | 40465 | 127 | 12 | 2.85 | 51 | Actin cytoplasmic type 5 [*Eg*] |
| NCBIprot | CDS26423.1 | 946 | 16412 | 16 | 3 | 1.13 | 37 | Actin protein 2:3 complex subunit 5 [*Hm*] |
| NCBIprot | CDS25545.1 | 232 | 37063 | 13 | 3 | 0.40 | 9 | Actin related protein 2:3 complex subunit 2 [*Hm*] |
| NCBIprot | AAA21482.1 | 5309 | 41645 | 118 | 12 | 3.11 | 49 | Actin, partial [*Dd*] |
| NCBIprot | CDS31513.2 | 321 | 69205 | 9 | 3 | 0.20 | 4 | ADP dependent glucokinase [*Hm*] |
| NCBIprot | CDS31970.1 | 1791 | 52632 | 43 | 10 | 1.23 | 28 | Alanine aminotransferase 2 [*Hm*] |
| NCBIprot | CDS26967.1 | 1182 | 34691 | 56 | 7 | 1.33 | 17 | Aldo keto reductase family 1 member B4 [*Hm*] |
| NCBIprot | CDS30937.1 | 186 | 74097 | 9 | 5 | 0.33 | 8 | Arginyl tRNA synthetase cytoplasmic [*Hm*] |
| NCBIprot | CDS28879.1 | 423 | 59655 | 17 | 5 | 0.42 | 10 | ATP synthase subunit alpha mitochondrial [*Hm*] |
| NCBIprot | CDS27086.1 | 1359 | 56141 | 35 | 9 | 0.96 | 25 | ATP synthase subunit beta mitochondrial [*Hm*] |
| NCBIprot | AEO79202.1 | 1327 | 41891 | 32 | 6 | 0.83 | 25 | Beta-tubulin, partial [*Hm*] |
| NCBIprot | CDS33663.2 | 180 | 87439 | 8 | 5 | 0.27 | 6 | Calpain [*Hm*] |
| NCBIprot | CDS29309.1 | 1254 | 39599 | 27 | 5 | 0.70 | 21 | Calponin [*Hm*] |
| NCBIprot | CDS32077.1 | 1138 | 192919 | 32 | 9 | 0.22 | 7 | Clathrin heavy chain [*Hm*] |
| NCBIprot | CDS29929.1 | 1890 | 37210 | 53 | 10 | 2.09 | 27 | Cytosolic malate dehydrogenase [*Hm*] |
| NCBIprot | CDS31349.1 | 447 | 39170 | 18 | 3 | 0.38 | 9 | Deoxyhypusine hydroxylase:monooxygenase [*Hm*] |
| NCBIprot | CDS29032.1 | 510 | 535841 | 8 | 6 | 0.05 | 2 | Dynein heavy chain [*Hm*] |
| NCBIprot | AAF13129.1 | 3603 | 29743 | 107 | 12 | 5.24 | 58 | Elongation factor 1 alpha, partial [*Hd*] |
| NCBIprot | CDS32105.1 | 270 | 94862 | 12 | 4 | 0.20 | 4 | Elongation factor 2 [*Hm*] |
| NCBIprot | CDS27698.1 | 420 | 22457 | 29 | 4 | 0.75 | 11 | Endophilin B1 [*Hm*] |
| NCBIprot | CDS30005.1 | 6699 | 48506 | 190 | 13 | 2.37 | 33 | Enolase [*Hm*] |
| NCBIprot | CDS34684.1 | 1072 | 48279 | 31 | 7 | 0.84 | 14 | Expressed protein [*Hm*] |
| NCBIprot | CDS27704.1 | 1093 | 15312 | 35 | 4 | 1.95 | 27 | Fatty acid binding protein a [*Hm*] |
| NCBIprot | CDS33172.1 | 2104 | 307771 | 50 | 14 | 0.21 | 7 | Filamin [*Hm*] |
| NCBIprot | CDS26447.1 | 12336 | 39864 | 227 | 13 | 2.94 | 56 | Fructose 16 bisphosphate aldolase [*Hm*] |
| NCBIprot | CDS31600.1 | 1145 | 62365 | 28 | 8 | 0.72 | 20 | Fumarate hydratase class I [*Hm*] |
| NCBIprot | AAP84347.1 | 1870 | 71553 | 31 | 4 | 0.27 | 6 | Glucose regulated protein GRP78 [*Se*] |
| NCBIprot | CDS29594.1 | 1914 | 58326 | 93 | 7 | 0.66 | 9 | Glutamate dehydrogenase [*Hm*] |
| NCBIprot | CDS25323.1 | 6968 | 55239 | 164 | 8 | 0.99 | 16 | Glutamate dehydrogenase mitochondrial [*Hm*] |
| NCBIprot | CDS33148.1 | 209 | 79295 | 7 | 4 | 0.24 | 7 | Glutamine:fructose 6 phosphate aminotransferase [*Hm*] |
| NCBIprot | CDS31614.1 | 16861 | 36715 | 234 | 9 | 1.80 | 31 | Glyceraldehyde 3 phosphate dehydrogenase [*Hm*] |
| NCBIprot | CDS19686.1 | 605 | 98186 | 25 | 6 | 0.29 | 7 | glycogen phosphorylase [*Eg*] |
| NCBIprot | CDS28178.2 | 3543 | 70873 | 54 | 11 | 0.93 | 24 | Heat shock protein 70 [*Hm*] |
| NCBIprot | CDS28182.1 | 1072 | 84308 | 26 | 7 | 0.42 | 9 | Heat shock protein 71 kDa protein [*Hm*] |
| NCBIprot | CDS27455.1 | 1796 | 72645 | 31 | 4 | 0.26 | 6 | Heat Shock protein family member (hsp 3) [*Hm*] |
| NCBIprot | CDI70178.1 | 515 | 74243 | 22 | 7 | 0.49 | 9 | Heat shock protein heat shock protein 90 alpha [*Eg*] |
| NCBIprot | CDS23579.1 | 449 | 53158 | 10 | 4 | 0.37 | 11 | Hexokinase [*Eg*] |
| NCBIprot | AAG09785.1 | 473 | 8678 | 17 | 3 | 3.08 | 35 | Hydrophobic ligand binding protein [*Hd*] |
| NCBIprot | XP_024351241.1 | 250 | 39885 | 9 | 3 | 0.37 | 11 | Hypothetical protein EGR_05043 [*Eg*] |
| NCBIprot | CUU99314.1 | 3581 | 61124 | 54 | 7 | 0.62 | 19 | Hypothetical transcript [*Hm*] |
| NCBIprot | CDS27386.1 | 274 | 32608 | 10 | 3 | 0.47 | 13 | Inorganic pyrophosphatase [*Hm*] |
| NCBIprot | CDS26883.1 | 603 | 36241 | 21 | 3 | 0.26 | 6 | Lactate dehydrogenase a [*Hm*] |
| NCBIprot | CDS29747.1 | 402 | 97767 | 7 | 3 | 0.14 | 6 | Major vault protein [*Hm*] |
| NCBIprot | F1C7I4.1 | 633 | 36770 | 21 | 3 | 0.41 | 7 | Malate dehydrogenase, cytoplasmic [*Ts*] |
| NCBIprot | CDS31809.1 | 311 | 40735 | 12 | 3 | 0.36 | 12 | Mitochondrial import receptor subunit tom34 [*Hm*] |
| NCBIprot | CDS33163.1 | 2203 | 224148 | 60 | 18 | 0.41 | 12 | Myosin heavy chain [*Hm*] |
| NCBIprot | CDS19483.1 | 590 | 228183 | 17 | 7 | 0.14 | 4 | Myosin heavy chain non muscle [*Eg*] |
| NCBIprot | CDS22215.1 | 1564 | 114070 | 34 | 11 | 0.50 | 15 | Na:K ATPase alpha subunit [*Eg*] |
| NCBIprot | CDS19779.1 | 485 | 48904 | 9 | 3 | 0.29 | 10 | NADP binding domain [*Eg*] |
| NCBIprot | CDS29227.2 | 1598 | 33380 | 53 | 3 | 0.46 | 9 | NADP dependent malic enzyme [*Hm*] |
| NCBIprot | CDS30121.1 | 417 | 40928 | 15 | 3 | 0.36 | 11 | Ndr [*Hm*] |
| NCBIprot | CDS25930.1 | 724 | 17276 | 24 | 4 | 1.05 | 22 | Nucleoside diphosphate kinase A [*Hm*] |
| NCBIprot | CDS27807.1 | 11095 | 70871 | 256 | 20 | 2.29 | 37 | Phosphoenolpyruvate carboxykinase [*Hm*] |
| NCBIprot | CDS16736.1 | 1061 | 63831 | 14 | 3 | 0.22 | 13 | Phosphoglucomutase [*Eg*] |
| NCBIprot | CDS32456.1 | 4393 | 44474 | 136 | 9 | 1.34 | 24 | Phosphoglycerate kinase 1 [*Hm*] |
| NCBIprot | CDS33978.1 | 1855 | 28784 | 56 | 9 | 2.71 | 45 | Phosphoglycerate mutase [*Hm*] |
| NCBIprot | CDS34495.1 | 258 | 30780 | 9 | 3 | 0.51 | 11 | Purine nucleoside phosphorylase [*Hm*] |
| NCBIprot | CDS17986.1 | 3714 | 62707 | 88 | 11 | 1.10 | 26 | Pyruvate kinase [*Eg*] |
| NCBIprot | CDS25961.1 | 4659 | 113844 | 97 | 12 | 0.56 | 16 | Pyruvate kinase isozymes M1:M2 [*Hm*] |
| NCBIprot | CDS29622.1 | 3211 | 282700 | 71 | 27 | 0.50 | 17 | Spectrin alpha actinin [*Hm*] |
| NCBIprot | CDS28968.2 | 1785 | 271653 | 49 | 17 | 0.30 | 10 | Spectrin beta chain [*Hm*] |
| NCBIprot | CDS32637.1 | 1156 | 71592 | 42 | 10 | 0.80 | 18 | Succinate dehydrogenase (ubiquinone) [*Hm*] |
| NCBIprot | CDS28334.1 | 907 | 34173 | 19 | 4 | 0.63 | 19 | Succinyl coenzyme A synthetase alpha subunit [*Hm*] |
| NCBIprot | CDS25937.1 | 645 | 83777 | 23 | 4 | 0.22 | 5 | Titin [*Hm*] |
| NCBIprot | CDS29022.1 | 787 | 88913 | 22 | 11 | 0.69 | 18 | Transitional endoplasmic reticulum atpase [*Hm*] |
| NCBIprot | CDS28549.1 | 953 | 68312 | 26 | 7 | 0.54 | 10 | Transketolase [*Hm*] |
| NCBIprot | CDS29962.1 | 2358 | 27806 | 69 | 9 | 3.49 | 38 | Triosephosphate isomerase [*Hm*] |
| NCBIprot | CDS29158.1 | 284 | 29480 | 8 | 3 | 0.53 | 15 | Troponin i [*Hm*] |
| NCBIprot | CDS34320.1 | 838 | 50714 | 31 | 3 | 0.28 | 9 | Tubulin alpha 1C chain [*Hm*] |
| NCBIprot | CDS26822.1 | 471 | 18093 | 18 | 3 | 0.99 | 20 | Ubiquitin conjugating enzyme E2 N [*Hm*] |
| NCBIprot | CDS25480.1 | 2384 | 38905 | 38 | 6 | 0.91 | 22 | UDP glucose 4 epimerase [*Hm*] |
| NCBIprot | CDS29466.1 | 1244 | 55940 | 31 | 4 | 0.35 | 6 | Viral IAP associated factor [*Hm*] |

*Dd* – *Diphyllobothrium* *dendriticum*; *Eg* – *Echinococcus* *granulosus*; *Hd* – *Hymenolepis* *diminuta*; *Hm* – *Hymenolepis* *microstoma*; *Se* – *Spirometra* *erinaceieuropaei;
Ts* – *Taenia solium*;
